# Supplementary material for: The causal relationship between the human gut microbiota and pyogenic arthritis: a Mendelian randomization study
Source: Front Cell Infect Microbiol. 2024 Nov 26;14:1452480. doi: 10.3389/fcimb.2024.1452480 (PMC11629706; doi:10.3389/fcimb.2024.1452480)
Supplement: Supplementary file 1 [file DataSheet1.zip › mendelian test/ebi-a-GCST90027512.csv_forest.pdf]

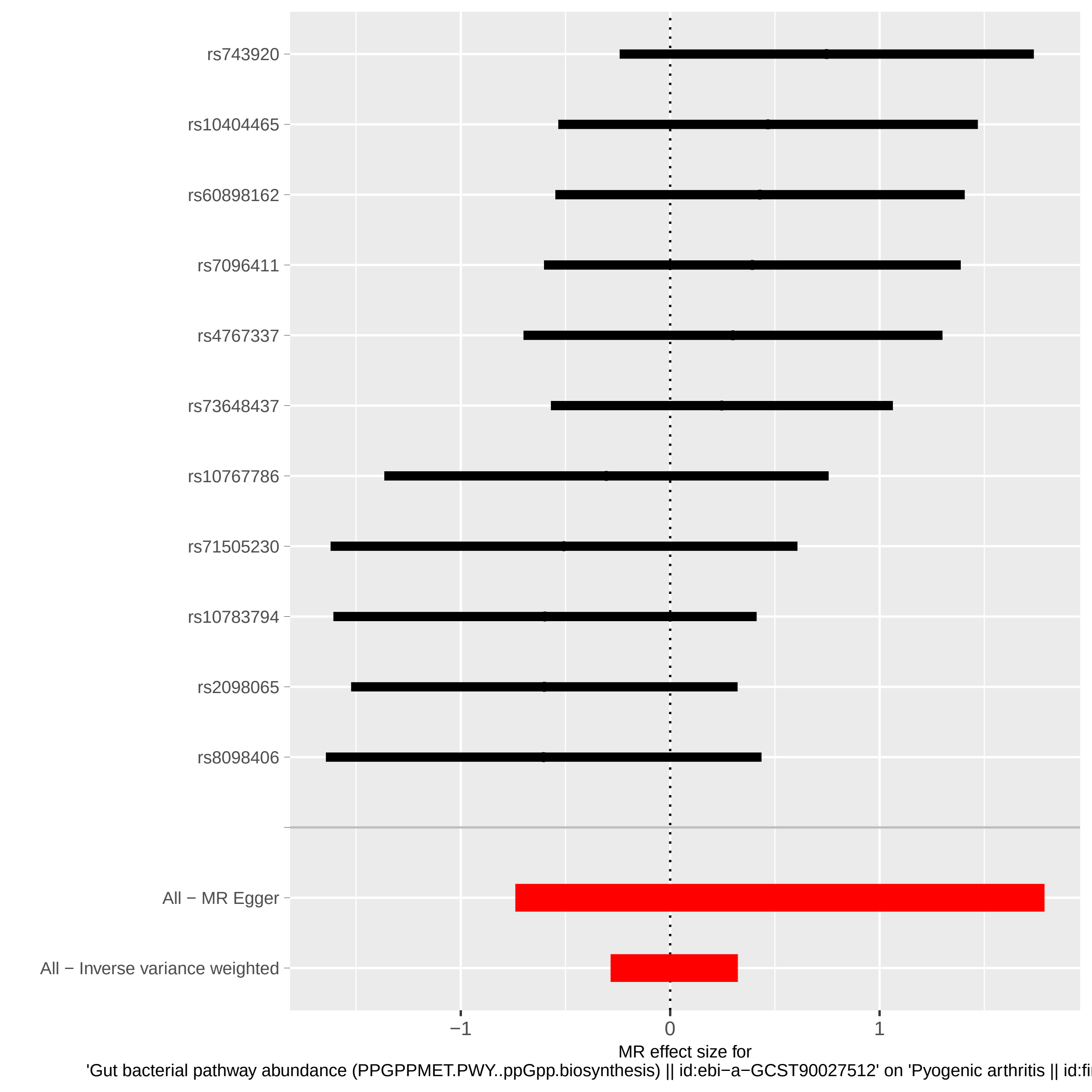
rs743920
rs10404465
rs60898162
rs7096411
rs4767337
rs73648437
rs10767786
rs71505230
rs10783794
rs2098065
rs8098406
All − MR Egger
All − Inverse variance weighted
−1
0
1
MR effect size for
'Gut bacterial pathway abundance (PPGPPMET.PWY..ppGpp.biosynthesis) || id:ebi−a−GCST90027512' on 'Pyogenic arthritis || id:fi
